# Ectopic ACTH-secreting pheochromocytoma without typical signs of Cushing syndrome

**DOI:** 10.1093/omcr/omaf005

**Published:** 2025-03-28

**Authors:** Zeltzin Soto-Montes, David Medina-Julio, Orlando D Solis-Coronado, Jesús G Mendoza-García, Erika K Tenorio-Aguirre, Froylan D Martínez-Sánchez

**Affiliations:** Department of Endocrinology, Instituto Nacional de Ciencias Medicas y Nutrición Salvador Zubiran, Vasco de Quiroga 15, Belisario Domínguez Secc 16, Tlalpan, Ciudad de Mexico 14080, Mexico; Department of Internal Medicine, Hospital General Dr. Manuel Gea Gonzalez, Calz. de Tlalpan 4800, Belisario Domínguez Secc 16, Tlalpan, Ciudad de Mexico 14080, Mexico; Facultad de Medicina, Universidad Nacional Autónoma de México, Escolar 411A, Copilco Universidad, Coyoacán, Ciudad de México 04360, Mexico; Division of Pathological Anatomy, Hospital General Dr. Manuel Gea Gonzalez, Calz. de Tlalpan 4800, Belisario Domínguez Secc 16, Tlalpan, Ciudad de Mexico 14080, Mexico; Department of Internal Medicine, Hospital General Dr. Manuel Gea Gonzalez, Calz. de Tlalpan 4800, Belisario Domínguez Secc 16, Tlalpan, Ciudad de Mexico 14080, Mexico; Department of Internal Medicine, Hospital General Dr. Manuel Gea Gonzalez, Calz. de Tlalpan 4800, Belisario Domínguez Secc 16, Tlalpan, Ciudad de Mexico 14080, Mexico; Department of Internal Medicine, Hospital General Dr. Manuel Gea Gonzalez, Calz. de Tlalpan 4800, Belisario Domínguez Secc 16, Tlalpan, Ciudad de Mexico 14080, Mexico; Facultad de Medicina, Universidad Nacional Autónoma de México, Escolar 411A, Copilco Universidad, Coyoacán, Ciudad de México 04360, Mexico

**Keywords:** Cushing syndrome, pheochromocytoma, ACTH

## Abstract

This case report describes a 42-year-old female with a rare pheochromocytoma presenting without classic Cushingoid features but with uncontrolled hypertension, type 2 diabetes, and recurrent headaches. Despite the absence of typical signs, biochemical analysis revealed elevated cortisol and ACTH levels, and imaging showed a 6 cm adrenal mass. The patient was stabilized preoperatively with alpha-blockers and metyrapone before undergoing a successful laparoscopic adrenalectomy. Histopathology confirmed pheochromocytoma with aggressive features. Postoperatively, her blood pressure and symptoms improved, and her cortisol levels normalized. This case underscores the diagnostic challenges of ACTH-secreting pheochromocytomas without classic hypercortisolism signs and emphasizes the need for thorough endocrine and imaging assessments. Surgical resection remains the definitive treatment, with long-term follow-up essential to monitor for recurrence. This case contributes to the limited literature on the coexistence of pheochromocytoma and ectopic ACTH secretion.

## Introduction

Ectopic ACTH-dependent tumors are rare, comprising approximately 5%–10% of Cushing syndrome cases, and are infrequently associated with pheochromocytomas, making this a unique presentation [1, 2]. Pheochromocytomas, though rare, can present as adrenal incidentalomas, often discovered during imaging for unrelated conditions. They represent 7% of adrenal incidentalomas and pose clinical challenges due to the risk of hormonal hypersecretion, including excess catecholamines and cortisol [1]. This case highlights the coexistence of an ectopic ACTH-producing tumor and pheochromocytoma, a combination rarely reported in the literature [3, 4]. While Cushing syndrome typically arises from adrenal or pituitary sources, ectopic ACTH secretion from pheochromocytomas presents a diagnostic and therapeutic challenge due to its rarity and aggressive potential [4–6]. Early diagnosis is crucial, particularly in cases with comorbidities like hypertension and diabetes, which are common in pheochromocytomas [1, 2]. This case underscores the need for a multidisciplinary approach to managing rare endocrine tumors.

## Case report

A 42-year-old female from Mexico City presented with a history of treatment-resistant hypertension and a newly identified adrenal mass. She had no history of alcohol or tobacco use and led a generally healthy lifestyle. She was diagnosed with type 2 diabetes five years before symptoms appeared and developed hypertension five years before hospitalization, managed with valsartan and amlodipine verapamil.

**Figure 1:**
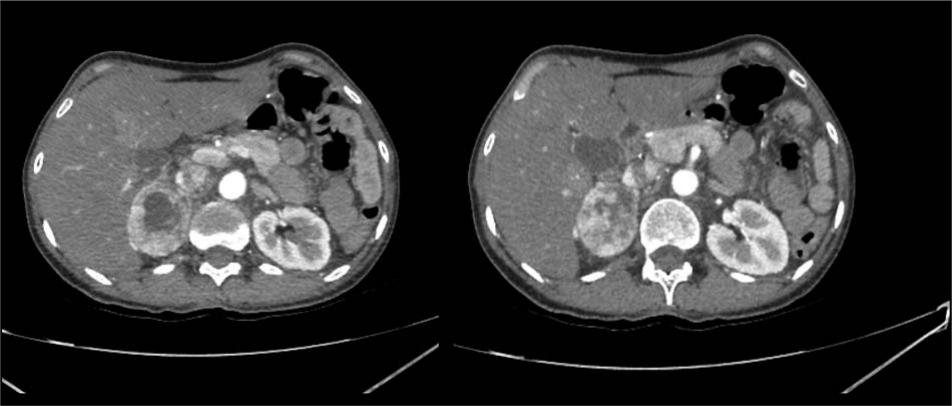
The imaging identified a hyperdense area at the lower pole of the left kidney. A heterogeneous image was visualized in the right adrenal gland, characterized by a hypodense lesion measuring 40 × 47 × 43 mm, with a density of 36 Hounsfield units (HU) in the simple phase, 107 HU in the venous phase and 61 HU in the delayed phase (15 min), with an absolute washout of 64%.

The patient’s hypertension worsened, with blood pressure readings reaching 200/160 mmHg. She presented with asthenia and adynamia, and a CT scan revealed a 4 cm right adrenal mass, confirmed as 4.7 cm on a subsequent scan (Fig. 1). No signs of metastasis were observed. Upon hospital admission, her physical examination revealed a blood pressure of 95/84 mmHg, a heart rate of 95 beats per minute, a respiratory rate of 28 breaths per minute, and a systolic murmur. She exhibited no Cushingoid features.

Initial laboratory tests showed elevated white blood cells (11 000/mm3), hemoglobin of 12.5 g/dl, and platelet count of 305 000/mm3. Blood chemistry indicated hyperglycemia (132 mg/dl), hyponatremia (129 mEq/l), and hypokalemia (3.4 mEq/l). Cortisol levels were elevated at 31.53 μg/dl, and a 1 mg low-dose dexamethasone suppression test showed cortisol levels of 16.65 μg/dl and 14.63 μg/dl, suggesting ACTH-dependent Cushing syndrome.

ACTH levels were 24 pg/ml, which, while elevated, were not suppressed. However, elevated 24-h urinary metanephrines (9881 μg/24 h) confirmed the presence of pheochromocytoma. The patient’s aldosterone-to-renin ratio was measured, revealing a ratio of 4. The serum aldosterone level was 5 ng/dl (138 pmol/l), while plasma renin activity was recorded at 1.1 ng/ml/h.

Imaging revealed a 4.7 cm right adrenal mass with a density of 36 Hounsfield Units and an absolute washout of 64%, with no signs of malignancy (Fig. 1).

The patient’s hypertension was initially managed with prazosin and metoprolol, but her blood pressure spiked to 200/160 mmHg during a hypertensive crisis, requiring nitroprusside. Surgical intervention was planned after diagnosis was confirmed.

**Figure 2:**
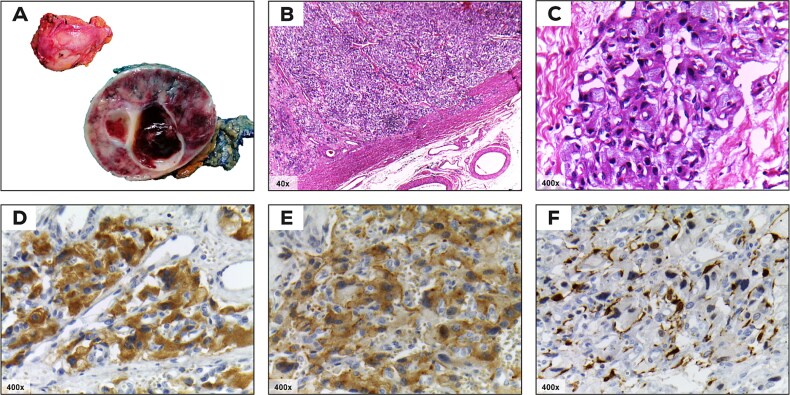
Histological and microscopic findings of adrenal Pheochromocytoma. (A) Macroscopic appearance. The ovoid tissue specimen has a light, smooth, soft external surface. The cut surface reveals a dark inner surface with light and hemorrhagic areas. Two cystic lesions with smooth walls are observed in the center (gross view). (B) A well-demarcated hypercellular lesion with an organoid pattern (Zellballen), separated by thin fibrovascular septa (Hematoxylin and eosin stain, 40×). (C) Nest of polygonal principal cells with ample eosinophilic granular cytoplasm, well-defined plasma membranes, hyperchromatic nuclei, and mild nuclear pleomorphism. Adjacent to the principal cells are spindle-shaped sustentacular cells with eosinophilic cytoplasm (Hematoxylin and eosin stain, 400×). (D) Positive immunoreactivity for chromogranin in principal cells. (E) Intense cytoplasmic reaction for synaptophysin in principal cells (immunohistochemistry, 400×). (F) Positive immunoreactivity for S100 protein, showing nuclear and cytoplasmic staining in sustentacular cells (immunohistochemistry, 400×).

The patient underwent a successful laparoscopic right adrenalectomy. The tumor measured 6 cm, and histopathology confirmed a pheochromocytoma with a PASS score of 4, indicating potential for aggressive behavior (Table 1). Histological and immunohistochemical analysis revealed the tumor’s characteristic organoid pattern (Zellballen) with chromogranin and synaptophysin positivity in principal cells and S100 protein staining in sustentacular cells, consistent with pheochromocytoma (Fig. 2). Postoperatively, her blood pressure stabilized, and symptoms of palpitations and sweating resolved. She has weaned off antihypertensives, and a follow-up dexamethasone suppression test showed a significant reduction in cortisol levels (1.2 μg/dl), indicating successful tumor removal.

**Table 1:** Histopathological report.

HISTOPATHOLOGICAL DIAGNOSIS
Specimen from right adrenalectomy: Pheochromocytoma measuring 6x6 cm (positive for chromogranin 7, synaptophysin +S100, with sustentacular cells staining positive) Marked nuclear pleomorphism: 1 point Diffuse growth pattern: 2 points Capsular invasion: 1 point
Total: 4 points.
Tumors with a score greater than 4 may exhibit aggressive biological behavior.

Postoperatively, her course was uneventful, with stable blood pressure without antihypertensives. A follow-up evaluation revealed normal cortisol levels, and 24-h urinary metanephrines returned to normal (312 μg/24 h for metanephrines; 225 μg/24 h for normetanephrines). Repeat imaging showed no residual adrenal mass. At her most recent follow-up, the patient remained asymptomatic with normal laboratory values, and no recurrence has been detected.

## Discussion

Ectopic ACTH-secreting pheochromocytomas are rare, accounting for a small percentage of ACTH-dependent Cushing syndrome cases [1, 4–6]. These tumors present diagnostic challenges, mainly when typical signs of Cushing syndrome, such as moon face, abdominal striae, or muscle weakness, are absent [3]. In this case, the patient exhibited only diabetes, uncontrolled hypertension, and recurrent headaches, symptoms that can also be attributed to pheochromocytoma itself [1]. The absence of Cushingoid features delayed the identification of ectopic ACTH secretion, making this case particularly difficult and unusual.

According to Gabi JN *et al*., most patients with ACTH-secreting pheochromocytomas present with severe hypercortisolism, including rapid weight gain and characteristic facial changes [3]. The absence of such features in this patient highlights the need to consider ectopic ACTH secretion in cases of adrenal masses, even without typical Cushing syndrome symptoms. This case illustrates how subtle presentations can lead to delayed diagnoses, emphasizing the importance of thorough evaluation in patients with adrenal tumors and metabolic abnormalities [1, 3].

The diagnostic approach for pheochromocytomas includes hormonal assays and imaging [7, 8]. Preoperative management for pheochromocytomas typically includes alpha-blockers to manage catecholamine excess [4, 7, 8]. This patient was managed with prazosin for blood pressure control and metyrapone to suppress cortisol production, consistent with clinical guidelines for managing ACTH-secreting tumors [5, 7, 8]. Despite the absence of Cushingoid features, careful preoperative preparation was essential to prevent complications during surgery.

Surgical resection is the definitive treatment for pheochromocytomas, particularly those secreting ACTH [8]. In this case, the patient underwent a successful laparoscopic adrenalectomy with no intraoperative complications. Histopathology confirmed a pheochromocytoma with marked nuclear pleomorphism and capsular invasion, suggesting potential aggressive behavior. Postoperatively, the patient’s blood pressure normalized, and her diabetes improved, aligning with outcomes reported in similar cases [4, 6]. Cortisol levels also returned to normal, demonstrating the effectiveness of adrenalectomy in resolving hypercortisolism.

A limitation in this case was the delayed recognition of ectopic ACTH secretion due to the absence of typical Cushingoid signs. The literature underscores the importance of considering this diagnosis, even in nonspecific cases [5].

Long-term management of pheochromocytomas, particularly those with aggressive features like capsular invasion, requires close follow-up [5, 7, 8]. Genetic testing should be considered, especially in patients with unusual presentations or family histories of endocrine disorders [1, 5]. Although not performed in this case, genetic testing could have provided further insight into the tumor’s etiology.
